# Supplementary material for: Measurement of Oxygen Transfer Rate and Specific Oxygen Uptake Rate of h-iPSC Aggregates in Vertical Wheel Bioreactors to Predict Maximum Cell Density Before Oxygen Limitation
Source: Bioengineering (Basel). 2025 Mar 22;12(4):332. doi: 10.3390/bioengineering12040332 (PMC12024368; doi:10.3390/bioengineering12040332)
Supplement: Supplementary file 1 [file bioengineering-12-00332-s001.zip › bioengineering-3516836-supplementary.pptx]

## Slide 1
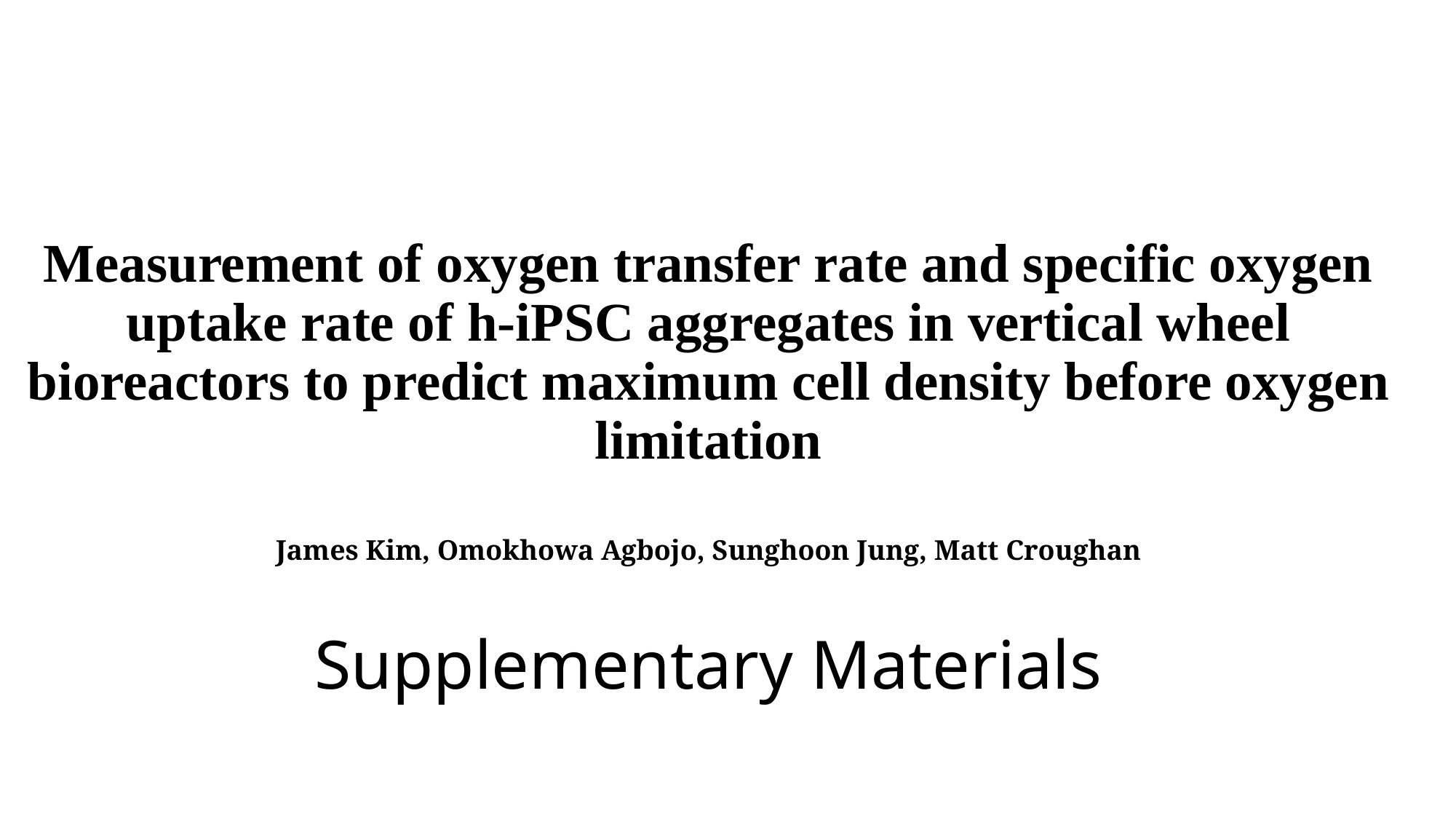

# Measurement of oxygen transfer rate and specific oxygen uptake rate of h-iPSC aggregates in vertical wheel bioreactors to predict maximum cell density before oxygen limitation James Kim, Omokhowa Agbojo, Sunghoon Jung, Matt Croughan Supplementary Materials

## Slide 2
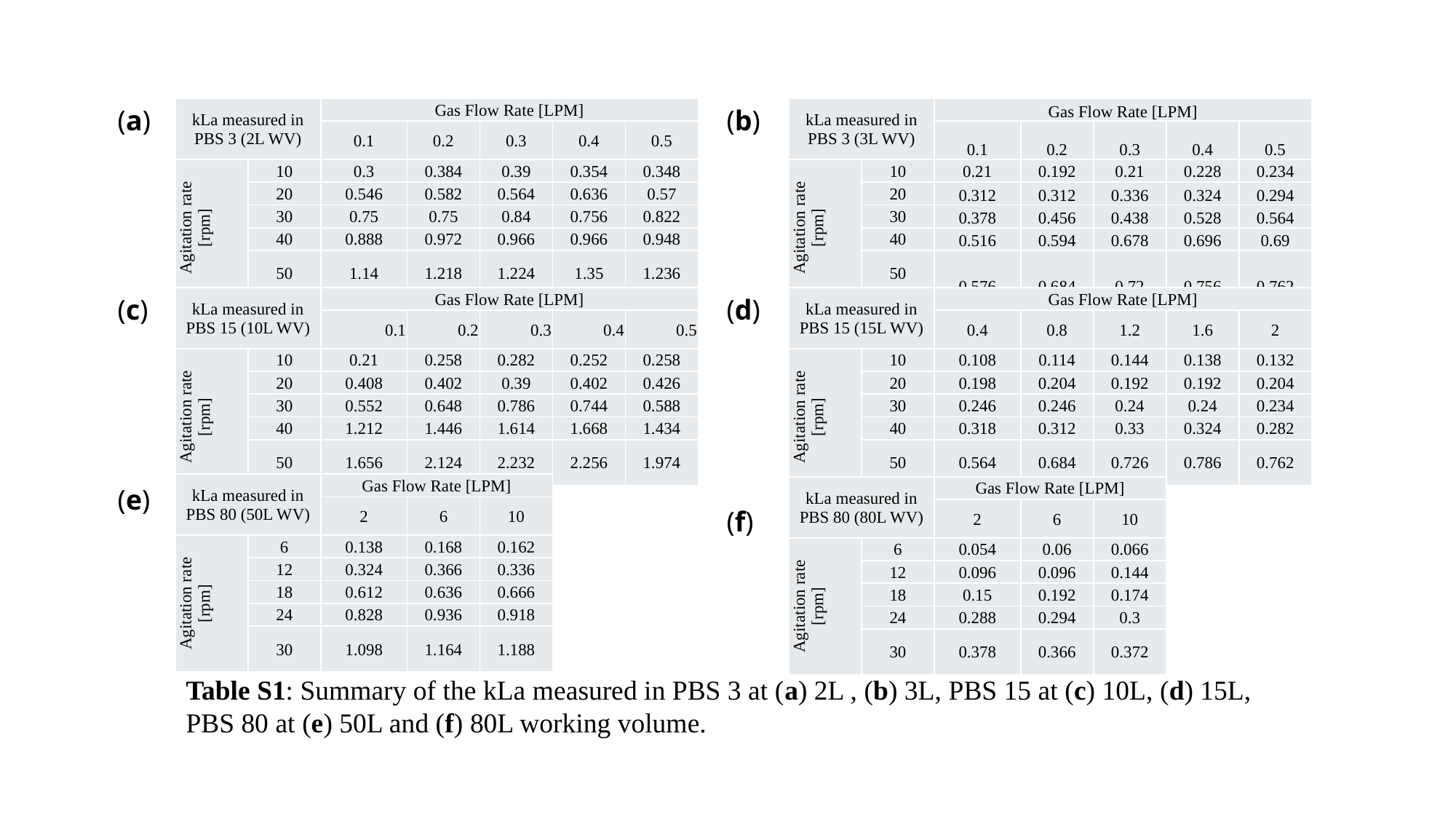

(a)
| kLa measured in PBS 3 (2L WV) | | Gas Flow Rate [LPM] | | | | |
| --- | --- | --- | --- | --- | --- | --- |
| | | 0.1 | 0.2 | 0.3 | 0.4 | 0.5 |
| Agitation rate [rpm] | 10 | 0.3 | 0.384 | 0.39 | 0.354 | 0.348 |
| | 20 | 0.546 | 0.582 | 0.564 | 0.636 | 0.57 |
| | 30 | 0.75 | 0.75 | 0.84 | 0.756 | 0.822 |
| | 40 | 0.888 | 0.972 | 0.966 | 0.966 | 0.948 |
| | 50 | 1.14 | 1.218 | 1.224 | 1.35 | 1.236 |
(b)
| kLa measured in PBS 3 (3L WV) | | Gas Flow Rate [LPM] | | | | |
| --- | --- | --- | --- | --- | --- | --- |
| | | 0.1 | 0.2 | 0.3 | 0.4 | 0.5 |
| Agitation rate [rpm] | 10 | 0.21 | 0.192 | 0.21 | 0.228 | 0.234 |
| | 20 | 0.312 | 0.312 | 0.336 | 0.324 | 0.294 |
| | 30 | 0.378 | 0.456 | 0.438 | 0.528 | 0.564 |
| | 40 | 0.516 | 0.594 | 0.678 | 0.696 | 0.69 |
| | 50 | 0.576 | 0.684 | 0.72 | 0.756 | 0.762 |
(c)
| kLa measured in PBS 15 (10L WV) | | Gas Flow Rate [LPM] | | | | |
| --- | --- | --- | --- | --- | --- | --- |
| | | 0.1 | 0.2 | 0.3 | 0.4 | 0.5 |
| Agitation rate [rpm] | 10 | 0.21 | 0.258 | 0.282 | 0.252 | 0.258 |
| | 20 | 0.408 | 0.402 | 0.39 | 0.402 | 0.426 |
| | 30 | 0.552 | 0.648 | 0.786 | 0.744 | 0.588 |
| | 40 | 1.212 | 1.446 | 1.614 | 1.668 | 1.434 |
| | 50 | 1.656 | 2.124 | 2.232 | 2.256 | 1.974 |
(d)
| kLa measured in PBS 15 (15L WV) | | Gas Flow Rate [LPM] | | | | |
| --- | --- | --- | --- | --- | --- | --- |
| | | 0.4 | 0.8 | 1.2 | 1.6 | 2 |
| Agitation rate [rpm] | 10 | 0.108 | 0.114 | 0.144 | 0.138 | 0.132 |
| | 20 | 0.198 | 0.204 | 0.192 | 0.192 | 0.204 |
| | 30 | 0.246 | 0.246 | 0.24 | 0.24 | 0.234 |
| | 40 | 0.318 | 0.312 | 0.33 | 0.324 | 0.282 |
| | 50 | 0.564 | 0.684 | 0.726 | 0.786 | 0.762 |
| kLa measured in PBS 80 (50L WV) | | Gas Flow Rate [LPM] | | |
| --- | --- | --- | --- | --- |
| | | 2 | 6 | 10 |
| Agitation rate [rpm] | 6 | 0.138 | 0.168 | 0.162 |
| | 12 | 0.324 | 0.366 | 0.336 |
| | 18 | 0.612 | 0.636 | 0.666 |
| | 24 | 0.828 | 0.936 | 0.918 |
| | 30 | 1.098 | 1.164 | 1.188 |
| kLa measured in PBS 80 (80L WV) | | Gas Flow Rate [LPM] | | |
| --- | --- | --- | --- | --- |
| | | 2 | 6 | 10 |
| Agitation rate [rpm] | 6 | 0.054 | 0.06 | 0.066 |
| | 12 | 0.096 | 0.096 | 0.144 |
| | 18 | 0.15 | 0.192 | 0.174 |
| | 24 | 0.288 | 0.294 | 0.3 |
| | 30 | 0.378 | 0.366 | 0.372 |
(e)
(f)
Table S1: Summary of the kLa measured in PBS 3 at (a) 2L , (b) 3L, PBS 15 at (c) 10L, (d) 15L, PBS 80 at (e) 50L and (f) 80L working volume.

## Slide 3
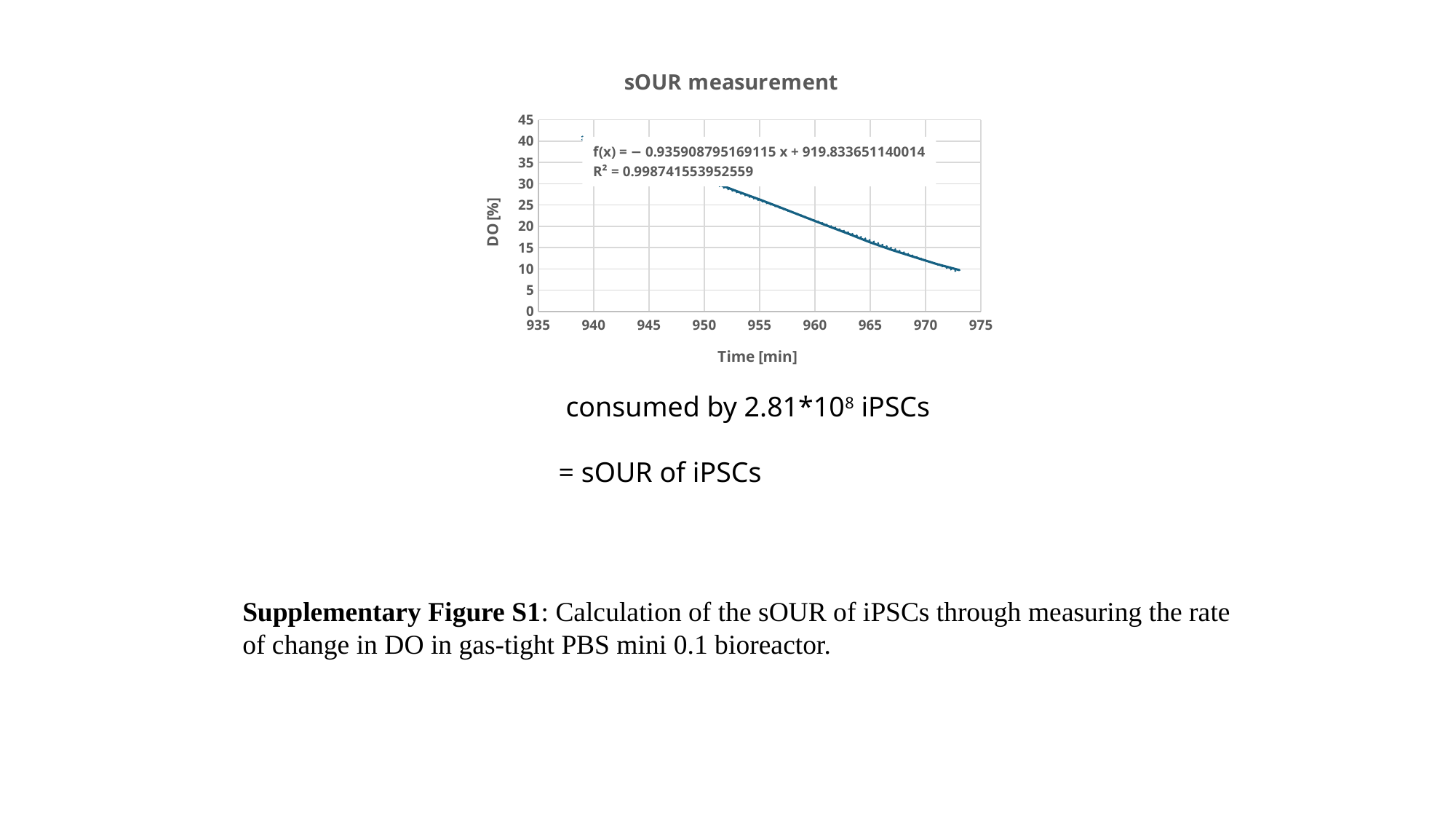

### Chart: sOUR measurement
| Category | |
|---|---|Supplementary Figure S1: Calculation of the sOUR of iPSCs through measuring the rate of change in DO in gas-tight PBS mini 0.1 bioreactor.

## Slide 4
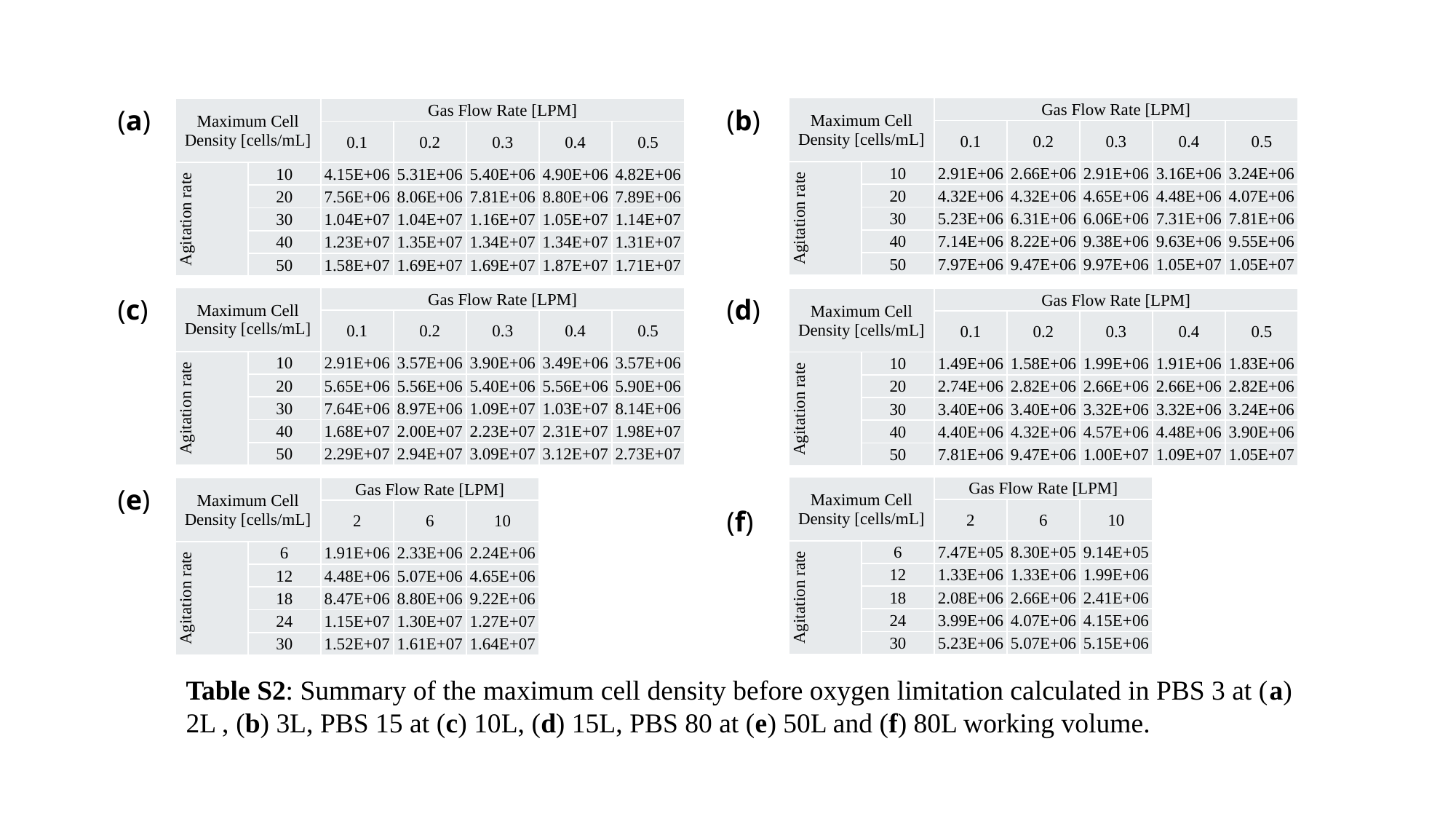

| Maximum Cell Density [cells/mL] | | Gas Flow Rate [LPM] | | | | |
| --- | --- | --- | --- | --- | --- | --- |
| | | 0.1 | 0.2 | 0.3 | 0.4 | 0.5 |
| Agitation rate | 10 | 2.91E+06 | 2.66E+06 | 2.91E+06 | 3.16E+06 | 3.24E+06 |
| | 20 | 4.32E+06 | 4.32E+06 | 4.65E+06 | 4.48E+06 | 4.07E+06 |
| | 30 | 5.23E+06 | 6.31E+06 | 6.06E+06 | 7.31E+06 | 7.81E+06 |
| | 40 | 7.14E+06 | 8.22E+06 | 9.38E+06 | 9.63E+06 | 9.55E+06 |
| | 50 | 7.97E+06 | 9.47E+06 | 9.97E+06 | 1.05E+07 | 1.05E+07 |
(a)
(b)
| Maximum Cell Density [cells/mL] | | Gas Flow Rate [LPM] | | | | |
| --- | --- | --- | --- | --- | --- | --- |
| | | 0.1 | 0.2 | 0.3 | 0.4 | 0.5 |
| Agitation rate | 10 | 4.15E+06 | 5.31E+06 | 5.40E+06 | 4.90E+06 | 4.82E+06 |
| | 20 | 7.56E+06 | 8.06E+06 | 7.81E+06 | 8.80E+06 | 7.89E+06 |
| | 30 | 1.04E+07 | 1.04E+07 | 1.16E+07 | 1.05E+07 | 1.14E+07 |
| | 40 | 1.23E+07 | 1.35E+07 | 1.34E+07 | 1.34E+07 | 1.31E+07 |
| | 50 | 1.58E+07 | 1.69E+07 | 1.69E+07 | 1.87E+07 | 1.71E+07 |
(c)
| Maximum Cell Density [cells/mL] | | Gas Flow Rate [LPM] | | | | |
| --- | --- | --- | --- | --- | --- | --- |
| | | 0.1 | 0.2 | 0.3 | 0.4 | 0.5 |
| Agitation rate | 10 | 2.91E+06 | 3.57E+06 | 3.90E+06 | 3.49E+06 | 3.57E+06 |
| | 20 | 5.65E+06 | 5.56E+06 | 5.40E+06 | 5.56E+06 | 5.90E+06 |
| | 30 | 7.64E+06 | 8.97E+06 | 1.09E+07 | 1.03E+07 | 8.14E+06 |
| | 40 | 1.68E+07 | 2.00E+07 | 2.23E+07 | 2.31E+07 | 1.98E+07 |
| | 50 | 2.29E+07 | 2.94E+07 | 3.09E+07 | 3.12E+07 | 2.73E+07 |
(d)
| Maximum Cell Density [cells/mL] | | Gas Flow Rate [LPM] | | | | |
| --- | --- | --- | --- | --- | --- | --- |
| | | 0.1 | 0.2 | 0.3 | 0.4 | 0.5 |
| Agitation rate | 10 | 1.49E+06 | 1.58E+06 | 1.99E+06 | 1.91E+06 | 1.83E+06 |
| | 20 | 2.74E+06 | 2.82E+06 | 2.66E+06 | 2.66E+06 | 2.82E+06 |
| | 30 | 3.40E+06 | 3.40E+06 | 3.32E+06 | 3.32E+06 | 3.24E+06 |
| | 40 | 4.40E+06 | 4.32E+06 | 4.57E+06 | 4.48E+06 | 3.90E+06 |
| | 50 | 7.81E+06 | 9.47E+06 | 1.00E+07 | 1.09E+07 | 1.05E+07 |
| Maximum Cell Density [cells/mL] | | Gas Flow Rate [LPM] | | |
| --- | --- | --- | --- | --- |
| | | 2 | 6 | 10 |
| Agitation rate | 6 | 7.47E+05 | 8.30E+05 | 9.14E+05 |
| | 12 | 1.33E+06 | 1.33E+06 | 1.99E+06 |
| | 18 | 2.08E+06 | 2.66E+06 | 2.41E+06 |
| | 24 | 3.99E+06 | 4.07E+06 | 4.15E+06 |
| | 30 | 5.23E+06 | 5.07E+06 | 5.15E+06 |
(e)
| Maximum Cell Density [cells/mL] | | Gas Flow Rate [LPM] | | |
| --- | --- | --- | --- | --- |
| | | 2 | 6 | 10 |
| Agitation rate | 6 | 1.91E+06 | 2.33E+06 | 2.24E+06 |
| | 12 | 4.48E+06 | 5.07E+06 | 4.65E+06 |
| | 18 | 8.47E+06 | 8.80E+06 | 9.22E+06 |
| | 24 | 1.15E+07 | 1.30E+07 | 1.27E+07 |
| | 30 | 1.52E+07 | 1.61E+07 | 1.64E+07 |
(f)
Table S2: Summary of the maximum cell density before oxygen limitation calculated in PBS 3 at (a) 2L , (b) 3L, PBS 15 at (c) 10L, (d) 15L, PBS 80 at (e) 50L and (f) 80L working volume.

## Slide 5
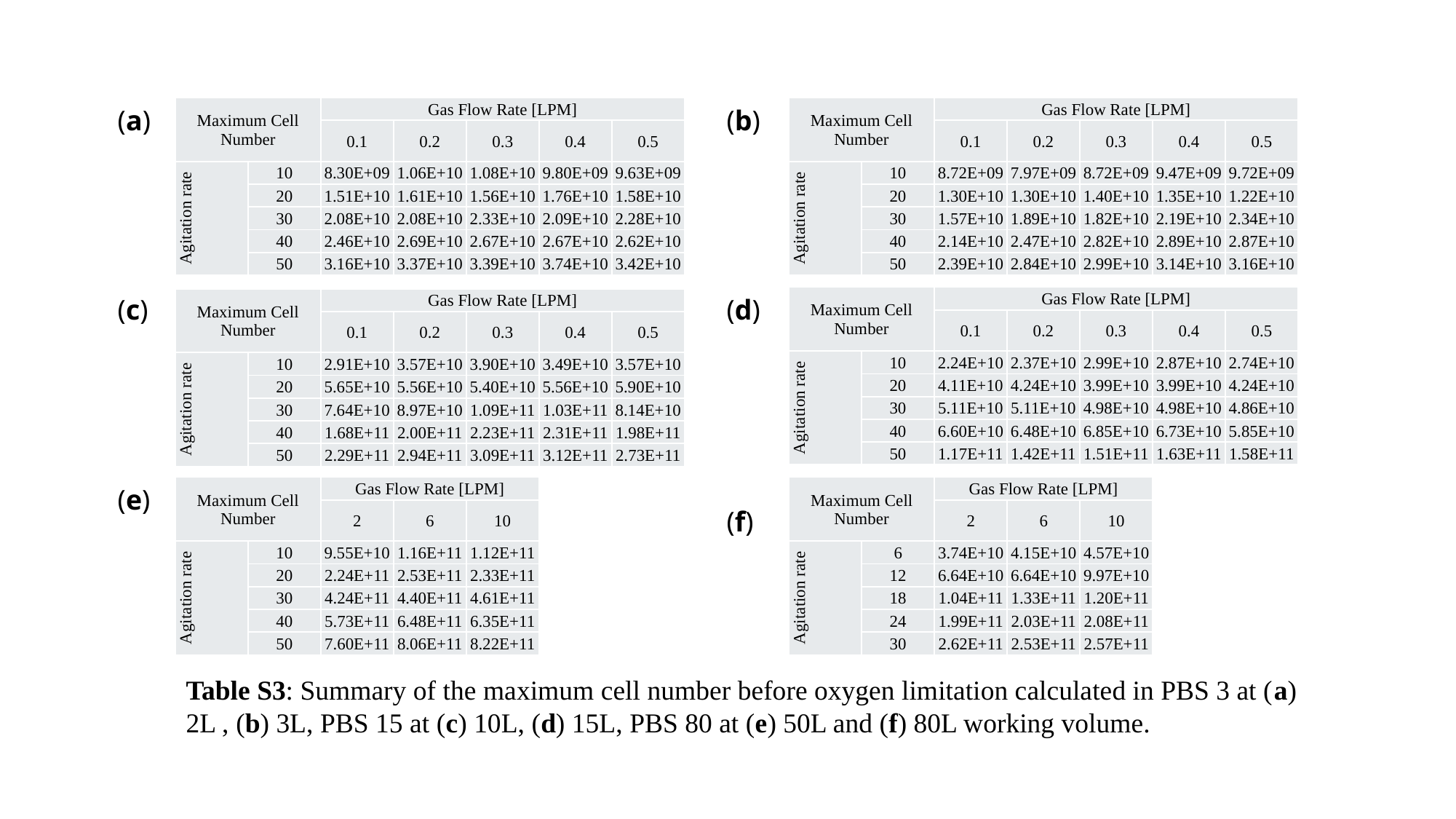

| Maximum Cell Number | | Gas Flow Rate [LPM] | | | | |
| --- | --- | --- | --- | --- | --- | --- |
| | | 0.1 | 0.2 | 0.3 | 0.4 | 0.5 |
| Agitation rate | 10 | 8.30E+09 | 1.06E+10 | 1.08E+10 | 9.80E+09 | 9.63E+09 |
| | 20 | 1.51E+10 | 1.61E+10 | 1.56E+10 | 1.76E+10 | 1.58E+10 |
| | 30 | 2.08E+10 | 2.08E+10 | 2.33E+10 | 2.09E+10 | 2.28E+10 |
| | 40 | 2.46E+10 | 2.69E+10 | 2.67E+10 | 2.67E+10 | 2.62E+10 |
| | 50 | 3.16E+10 | 3.37E+10 | 3.39E+10 | 3.74E+10 | 3.42E+10 |
| Maximum Cell Number | | Gas Flow Rate [LPM] | | | | |
| --- | --- | --- | --- | --- | --- | --- |
| | | 0.1 | 0.2 | 0.3 | 0.4 | 0.5 |
| Agitation rate | 10 | 8.72E+09 | 7.97E+09 | 8.72E+09 | 9.47E+09 | 9.72E+09 |
| | 20 | 1.30E+10 | 1.30E+10 | 1.40E+10 | 1.35E+10 | 1.22E+10 |
| | 30 | 1.57E+10 | 1.89E+10 | 1.82E+10 | 2.19E+10 | 2.34E+10 |
| | 40 | 2.14E+10 | 2.47E+10 | 2.82E+10 | 2.89E+10 | 2.87E+10 |
| | 50 | 2.39E+10 | 2.84E+10 | 2.99E+10 | 3.14E+10 | 3.16E+10 |
(a)
(b)
| Maximum Cell Number | | Gas Flow Rate [LPM] | | | | |
| --- | --- | --- | --- | --- | --- | --- |
| | | 0.1 | 0.2 | 0.3 | 0.4 | 0.5 |
| Agitation rate | 10 | 2.24E+10 | 2.37E+10 | 2.99E+10 | 2.87E+10 | 2.74E+10 |
| | 20 | 4.11E+10 | 4.24E+10 | 3.99E+10 | 3.99E+10 | 4.24E+10 |
| | 30 | 5.11E+10 | 5.11E+10 | 4.98E+10 | 4.98E+10 | 4.86E+10 |
| | 40 | 6.60E+10 | 6.48E+10 | 6.85E+10 | 6.73E+10 | 5.85E+10 |
| | 50 | 1.17E+11 | 1.42E+11 | 1.51E+11 | 1.63E+11 | 1.58E+11 |
(c)
(d)
| Maximum Cell Number | | Gas Flow Rate [LPM] | | | | |
| --- | --- | --- | --- | --- | --- | --- |
| | | 0.1 | 0.2 | 0.3 | 0.4 | 0.5 |
| Agitation rate | 10 | 2.91E+10 | 3.57E+10 | 3.90E+10 | 3.49E+10 | 3.57E+10 |
| | 20 | 5.65E+10 | 5.56E+10 | 5.40E+10 | 5.56E+10 | 5.90E+10 |
| | 30 | 7.64E+10 | 8.97E+10 | 1.09E+11 | 1.03E+11 | 8.14E+10 |
| | 40 | 1.68E+11 | 2.00E+11 | 2.23E+11 | 2.31E+11 | 1.98E+11 |
| | 50 | 2.29E+11 | 2.94E+11 | 3.09E+11 | 3.12E+11 | 2.73E+11 |
| Maximum Cell Number | | Gas Flow Rate [LPM] | | |
| --- | --- | --- | --- | --- |
| | | 2 | 6 | 10 |
| Agitation rate | 10 | 9.55E+10 | 1.16E+11 | 1.12E+11 |
| | 20 | 2.24E+11 | 2.53E+11 | 2.33E+11 |
| | 30 | 4.24E+11 | 4.40E+11 | 4.61E+11 |
| | 40 | 5.73E+11 | 6.48E+11 | 6.35E+11 |
| | 50 | 7.60E+11 | 8.06E+11 | 8.22E+11 |
| Maximum Cell Number | | Gas Flow Rate [LPM] | | |
| --- | --- | --- | --- | --- |
| | | 2 | 6 | 10 |
| Agitation rate | 6 | 3.74E+10 | 4.15E+10 | 4.57E+10 |
| | 12 | 6.64E+10 | 6.64E+10 | 9.97E+10 |
| | 18 | 1.04E+11 | 1.33E+11 | 1.20E+11 |
| | 24 | 1.99E+11 | 2.03E+11 | 2.08E+11 |
| | 30 | 2.62E+11 | 2.53E+11 | 2.57E+11 |
(e)
(f)
Table S3: Summary of the maximum cell number before oxygen limitation calculated in PBS 3 at (a) 2L , (b) 3L, PBS 15 at (c) 10L, (d) 15L, PBS 80 at (e) 50L and (f) 80L working volume.
